# Persistent Postpartum Pain After Elective Cesarean Section Is Not Only Persistent Postsurgical Pain—A Retrospective Study

**DOI:** 10.3390/healthcare13243282

**Published:** 2025-12-15

**Authors:** Agata Michalska, Daniel Wolder, Anna Błażuk-Fortak, Aleksandra Gładyś-Jakubczyk, Michał Błażuk, Justyna Pogorzelska, Anna Zmyślna, Waldemar Brola, Grzegorz Świercz

**Affiliations:** 1 Collegium Medicum, Jan Kochanowski University in Kielce, IX Wieków Kielc 19a, 25-516 Kielce, Poland; daniel.wolder@ujk.edu.pl (D.W.);; 2 Clinic of Obstetrics and Gynecology, Provincial Combined Hospital in Kielce, Grunwaldzka 45, 25-736 Kielce, Poland; 3 D. Karol Jonscher Municipal Medical Center, Milionowa 14, 93-113 Lodz, Poland

**Keywords:** persistent pain, postpartum pain, postsurgical pain, back pain, cesarean section

## Abstract

**Background**: Persistent postpartum pain (PPP) is a common condition after cesarean section (CS) that affects multiple domains of quality of life. PPP was defined as pain of any cause (not only related to surgery) appearing after CS and persisting for at least the three following months. The objective of this study was to calculate the incidence of PPP in women after elective CS and to analyze the associated risk factors. **Methods**: It was a retrospective cross-sectional study. An electronic patient-reported outcome tool (ePRO) was used to assess patients’ perception of their own health and to assess the presence and severity of pain. **Results**: Pain during pregnancy was reported by 66.14% of the study group. The most frequently reported localizations of pain were the lumbar spine, pubic symphysis, lower limbs, and sacrococcygeal region. The mean postoperative pain (day 0) defined by a Numeric Rating Scale was 5.44 (2.78 SD), and on the day of discharge (usually the third day after CS), it was 3.6 (2.29 SD). PPP occurred in 32.37% of women, was usually mild in nature, and had a little to moderate impact on function. Previous surgery raises the odds of PPP by 48.7% (OR = 1.487), pain during pregnancy raises the odds of PPP by 48.1% (OR = 1.481), and each additional point of the NRS on day 0 raises the odds of PPP by 16.6% (OR = 1.166). **Conclusions**: A higher risk of PPP could be found in women with pre-existing pain conditions, previous surgery, and severe postoperative pain. Persistent postpartum pain is not limited only to the area of surgery. Persistent back pain was reported by every second woman with PPP.

## 1. Introduction

According to the World Health Organization (WHO), cesarean section (CS) rates have been steadily increasing in low-, middle-, and high-income countries above levels that cannot be considered medically necessary [1]. CS may have short-term and long-term effects on the mother. Commonly reported short-term effects are postpartum hemorrhage, surgical site infection, puerperal fever, wound dehiscence, respiratory tract infection, anemia, reactions to anesthesia, blood clots, surgical injury, anxiety, and depression. Increased risk of placenta previa, placenta accreta, and placental abruption in subsequent pregnancies, increased risk of miscarriage, uterine rupture risk, pelvic floor dysfunction, chronic pain, and adhesions are commonly reported long-term effects. Compared with women who had a vaginal birth, women after a CS were more likely to report extreme tiredness and back pain. Breastfeeding problems, pain-related worsened sleep quality and comfort, delayed recovery, and prolonged hospitalization are also mentioned [2,3,4,5].

Cesarean section is associated with a high prevalence of pain conditions, such as postsurgical pain, pain onsetting during pregnancy and continuing postpartum, pain onset related to postpartum musculoskeletal changes and lifestyle changes (including newborn care), as well as anxiety and/or fatigue. Low back pain, pelvic girdle pain, de Quervain’s tenosynovitis, carpal tunnel syndrome, meralgia paresthetica, plantar fasciitis, and thoracic outlet syndrome are pain conditions reported in pregnancy. Most of these can also persist or occur in the postpartum period [6]. Postoperative pain is a complex physiological response to tissue injury accompanying surgical manipulation. It has the character of acute somatic and/or visceral pain arising due to tissue trauma (interruption of superficial tissues—skin, subcutaneous tissue, fascia, and muscles—and deeper structures/organs such as the peritoneum and uterus) and stretching of visceral structures. The postoperative pain after CS is severe, but it is self-limiting in nature. Postoperative pain resolves in two phases, with an initial exponential decline followed by a linear pattern [7,8,9]. The duration of postpartum pain has not been established yet and ranges from two to even up to six months [8,10]. The recovery process is reported to be individually variable, ranging from very fast resolution of pain (6 days) to much slower courses (>40 days) [7]. Pain that persists beyond the healing process is referred to as persistent pain. The precise definition of persistent postpartum pain (PPP) has not been developed yet. It is assumed that PPP lasts at least six weeks after childbirth. PPP following CS may be considered chronic postsurgical pain, which is defined as follows: (1) pain developing or increasing in intensity after CS, (2) pain persisting beyond the healing process (at least 3 months after the initiating event), (3) pain interfering with the quality of life, (4) pain located in the area of injury, projected to the territory of a nerve situated in this area, or referred to a dermatome [10,11].

When estimating the prevalence of persistent postoperative pain, pre-existing pain conditions should be excluded. It is difficult to separate pregnancy and postpartum pain conditions; hence, adequate assessment of postoperative persistent pain and PPP prevalence remains a challenge for researchers. For the purposes of the study, a definition of PPP was adopted based on the definition of postoperative pain excluding only the point relating to the pain localization (pain beyond the area of surgery trauma was also included as women were often reporting other types of pain onset connected to CS). The following pain was considered PPP:

- Localization: any pain, not only in the surgical field or projected to deep somatic or visceral tissues (musculoskeletal pain included);
- Duration: persisting for at least three months after surgery;
- Lowering the quality of life;
- Not present before CS.

The aim of this study was to evaluate the long-term postpartum pain experience in the group of women undergoing elective cesarean section. An electronic patient-reported outcome tool (ePRO) was used to assess patients’ perception of their own health, including aspects like daily functioning, as well as to determine the presence and severity of pain.

## 2. Materials and Methods

### 2.1. Participants

The participants were women after elective transperitoneal cesarean section with transverse skin incision. In order to minimize disruptive factors, such as the course of labor before CS or urgent medical conditions, for example, placental abruption, that may interfere with pain perception, emergency CSs were excluded.

### 2.2. Procedures

It was a retrospective cross-sectional study approved by the Jan Kochanowski University’s Bioethics Committee (12/2021). The study was conducted in the form of an online survey addressed to women who had undergone a CS. Information about the possibility of participation was posted on the official Facebook page of the Provincial Combined Hospital in Kielce, implementing the project. The website post contained a description of the purpose of the study, the conditions of participation, and a link redirecting to the survey platform. Participation in the study was voluntary and anonymous. After accessing the questionnaire, participants had to read the information about the project and give their informed consent to participate. In addition, the authors supported recruitment by sharing information about the study via social media, which broadened the reach of the message. Only women living in Poland were asked to participate in the study, as the survey was in Polish. Due to the recruitment online and the lack of a requirement to provide an exact location, it was not possible to determine precisely which regions of the country the respondents came from. The time taken to complete the questionnaire was approximately 20 min. In the case of a repeated CS, the answers should have referred to the last surgery. All answers were mandatory (without answering a question, it was not possible to move on to the next one), which prevented data loss.

The data analysis consisted of two stages: Stage I—analysis of the entire study group (the course and nature of pain during pregnancy, the severity of postoperative pain, and the course of recovery in all participants were assessed). The aim of this stage was to describe the full pain profile in the population of women after CS. Stage II was focused on identification of a subgroup meeting the criteria for PPP. In this subgroup, the relationships between PPP and pain during pregnancy, postoperative pain, and the course of recovery were examined.

### 2.3. Measures

The main outcomes constituted the experience of pain during pregnancy, after CS (postoperative pain), and in the postpartum period (persistent postpartum pain). The severity, duration, localization, and characteristics of pain were analyzed. The presence of pain was defined by a Numeric Rating Scale score > 0. A 4, 7 cut-off point scheme was used [12]. This theory presumes that mild pain is scored 4 or lower and moderate to severe pain 5 or higher. After preliminary assessment of pain during pregnancy, postoperative pain, and recovery (Stage I), a group of patients meeting the PPP criteria was included in the next stage of analysis (Stage II).

Participants were asked questions regarding their

- Reproductive and pregnancy background;
- Hospitalization (level of referral system, length of hospitalization);
- CS-related information (type of anesthesia, type of sutures, drainage);
- Newborn-related information (early skin-to-skin contact—contact during the first hour of a newborn’s life with the mother or father, newborn feeding method);
- Pregnancy, convalescence, and postpartum pain experience (pain during movement/activity was assessed, severity—using a Numeric Rating Scale, duration—in weeks, localization, characteristics);
- Pain-increasing and decreasing activities;
- Current health status (self-rated health, continuous numeric variable with a range of 0–100);
- Impact of pain on daily activities, physical activity, sleep, sexual intercourse, and childcare (self-rated, qualitative variables: no impact, little, moderate, and significant impact, prevents performance);
- Physiotherapy during pregnancy and in the postpartum period.

### 2.4. Statistical Analysis

The mean, standard deviation, median, quartiles, and range of quantitative variables were shown. For qualitative variables, absolute and relative frequencies (n and %) were reported. The chi-squared test (with Yates correction for 2 × 2 tables) or Fisher’s exact test (in case of low expected values) were used for comparisons of qualitative variables between groups. The Mann–Whitney U test was used for comparisons of quantitative variables between two groups, while the Kruskal–Wallis test (followed by the post hoc Dunn test) was used for three or more groups. Spearman’s correlation coefficient was used to assess correlation between two quantitative variables. Multiple linear regression was employed to model the potential impact of predictors on a quantitative variable. The regression parameters, alongside the 95% confidence intervals, were presented. Univariate and multiple logistic regression was employed to model the potential impact of predictors on a dichotomous variable. ORs (odds ratios), alongside the 95% confidence intervals, were presented. The significance level was set to 0.05. All the analyses were conducted in R software, version 4.5.0.

## 3. Results

**Figure 1:**
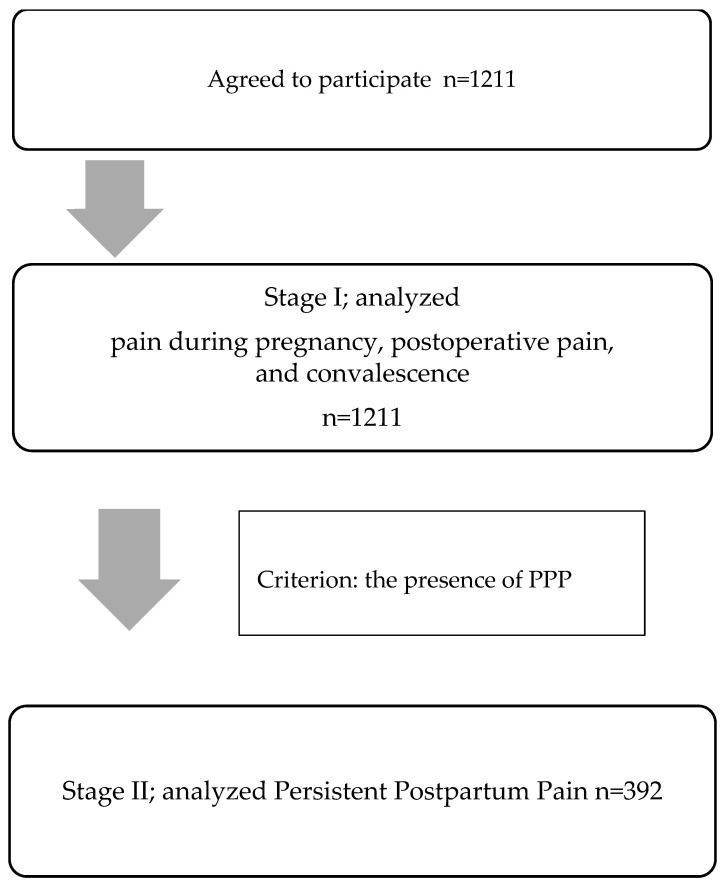
Selection process flow chart.

Data from 1211 patients were evaluated for this study (Figure 1). Characteristics of the study group can be found in Table 1. The mean age of the women was 32.51 (4.22 SD) with a range of 21 to 47 years old. Just over half of the study group had one cesarean section, and one third had a gynecological or abdominal operation before the CS. The mean postoperative period duration was 25.24 months (25.8 SD) with less than 12 months in almost half of the subjects. The hospital stay of half of the women was standard, and the reason for the prolonged stay was more often the health of the newborn than maternal complications. Spinal anesthesia predominated in the study group. Both nonabsorbable and absorbable sutures were used (46.90% vs. 53.10%). Wound drainage was used in almost 15% of the subjects.

### 3.1. Pregnancy Pain

Pain during pregnancy was reported by 66.14% of the women in the study group, and 31.54% underwent physiotherapy treatment for this reason. The most frequently reported localizations of pain were the lumbar spine (41.95%), pubic symphysis (24.44%), lower limbs (16.27%), and sacrococcygeal region (15.77%). The mean number of pain areas was 1.37 (1.37 SD) with a range of 0 to 7 (Table 2). The most common co-occurring areas were the lumbar spine and pubic symphysis (171/1211), lumbar spine and lower limbs (127/1211), lumbar spine and sacrococcygeal region (115/1211), lumbar spine and anal region (93/1211), pubic symphysis and lower limbs (86/1211), pubic symphysis and anal region (78/1211), pubic symphysis and pelvic floor (77/1211), and pubic symphysis and sacrococcygeal region (74/1211).

### 3.2. Postoperative Pain

The mean postoperative pain after CS (day 0) in the study group was 5.44 (2.78 SD) with a range from 0 to 10, and on the day of discharge (usually the third day after CS) it was 3.6 (2.29 SD) with the same range from 0 to 10. Referring to the intensity on the NRS scale, postoperative pain can be described as moderate pain decreasing to mild on the day of discharge. The mean pain decrease (discharge vs. day 0) was 1.84 points on the NRS scale (2.84 SD). The most common pain localization after CS was the postoperative wound (88.69%), abdomen (47.23%), and lumbar spine (15.19%), and less often, the ribs and shoulder. The mean number of pain localization was 1.7 (0.79 SD) with a range of 0 to 7. The most significant pain-increasing activities were verticalization, changing position in bed, and breastfeeding. Postoperative pain limited self-care and caring for the newborn in the majority of women (Table 3). In univariate analysis, there were no statistically significant differences in postoperative pain on day 0 by age, number of cesarean sections, previous operations, type of anesthesia, type of sutures, use of wound drainage, or presence of surgical site infection (SSI). Significant differences in postoperative pain intensity on day 0 but not on the day of discharge according to the level of referral system were found (primary level 5.91/2.7 SD; secondary level 5.17/2.86 SD, level tertiary 5.4/2.72 SD; *p* = 0.014, primary level > secondary and tertiary level). Postoperative pain reported on day 0 and on the day of discharge was significantly higher in women who experienced pain during pregnancy (Table 4). A multivariate linear regression model showed that when survey responders reported pain during pregnancy, they evaluated pain on day 0 as stronger by an average of 0.668. Moreover, CS at a secondary care level was considered to reduce pain on day 0 by an average of 0.767 and at a tertiary care level by an average of 0.594 compared with delivery at a primary care level (Table 5).

### 3.3. Persistent Postpartum Pain

According to the definition adopted in the study, PPP was present in 392/1211 women, which accounted for 32.37% of the study population (Table 6). Taking into consideration postoperative period duration, the rate of PPP was comparable in groups, ranging from 26.88% to 33.52% (Table 7). The mean PPP intensity was 3.44 (1.83 SD). Mild pain intensity (NRS score 1–4) was reported most frequently, regardless of the postoperative period duration. The rate of women reporting moderate and severe pain was lower, 21.17% (83/392) and 3.32% (13/392), respectively (Table 8). The most common pain localizations were the lumbar spine (48.72%), postoperative scar (44.13%), and abdomen (27.81%), and less often, the head, pelvis, and groin. The mean number of persistent pain localizations was comparable to the number of pain areas during early recovery (1.53/0.7 SD; with a range of 1 to 5). The postoperative wound and abdomen (49/392), postoperative wound and lumbar spine (41/392), and abdomen and lumbar spine (30/392) were most often indicated together. These three localizations of pain were mentioned most frequently, regardless of the postoperative period duration (Table 9).

On a scale of 0 to 100, women in the study group rated their current health status at 76.82 (18.63 SD). Only 11.48% of study group rated their health below 50. The group reporting the presence of PPP had significantly lower self-rated health (80.43/16.74 SD vs. 69.29/20.08 SD) (Table 10). Significant impact of pain on activity or inability to carry out activity occurred in 20/392 women in the area of the daily activities, 47/392 in the area of physical activity, 25/392 in the area of sleep quality, 53/392 in the area of sexual intercourse, and 9/392 in the area of childcare. Lifting of objects and physical activity were activities which exacerbated the PPP (Table 6).

Univariate logistic regression models (separate for each variable considered) showed that a previous surgery raises the odds of PPP by 49.7% (OR = 1.497), pain during pregnancy raises the odds of PPP by 62.2% (OR = 1.622), and each additional point of the NRS scale on day 0 raises the odds of PPP by 7.6% (OR = 1.076). A multivariate logistic regression model showed that a previous surgery raises the odds of PPP by 48.7% (OR = 1.487) and pain during pregnancy raises the odds of PPP by 48.1% (OR = 1.481). Each additional point of the NRS scale on day 0 raises the odds of PPP by 16.6% (OR = 1.166), whereas decreasing pain intensity by each additional point of the NRS scale decreases the odds of PPP by 12.1% (OR = 0.879). Hospitalization prolonged due to the child’s condition increases the odds of PPP by 52.1% (OR = 1.521) compared to standard hospitalization (Table 11).

## 4. Discussion

Although demographic and socio-economic factors are mainly cited as the causes of low fertility, many countries are implementing programs aimed at improving the quality of pre-, intra-, and postnatal care in order to reverse this trend. The issue of acute pain assessment and management is an important part of these programs. The problem of persistent postpartum pain and quality of postpartum life are also analyzed. The reported incidence of PPP varies, depending on the study population, study design, and criteria used; hence, the results of studies are inconsistent [9,13]. The context of persistent pain after CS remains a very specific condition. As with any surgical procedure, the degree of pain experienced by the patient is influenced by the location of the procedure (transperitoneal vs. extraperitoneal CS), its extent, the degree of tissue trauma, and psychogenic factors: anxiety and fear of experiencing pain [8,9,13]. In terms of severity, pain after a CS ranks 9th out of 179 different surgical procedures [14]. The probability of persistent postoperative pain in the adult population is approximately 20% [13]. Women are more likely to experience severe, persistent pain after surgery [5,8]. Data on the incidence of PPP after CS, compared to chronic pain in women after abdominal and gynecological surgery, are inconsistent. Studies showing a similar incidence emphasize the common mechanism underlying this phenomenon [15]. In the case of lower rates of persistent pain after CS, shorter operation times, less peripheral nerve damage, high rates of spinal anesthesia, and the protective role of oxytocin, estrogen, and progesterone are discussed [5,9].

After CS the scar pain/wound-site pain and visceral pain (deep intra-abdominal pain, pelvic pain) may co-exist with non-wound pain: low back pain, pain in the genito-pelvic region, or musculoskeletal pain occurring after surgery. For the purpose of our own study, PPP was defined as pain of any cause (not only related to surgery) persisting for at least three months after CS. Similar assumptions were made by Daly et al. [16] and Jin et al. [17]. In the present study, the incidence of PPP was considered high (32.37%), assuming that the likelihood of developing persistent postsurgical pain is approximately 20% or even lower than 10% [8,13,15]. In a British study incidence of new pain at four months was 35.7% and 41.8% in the group that reported preoperative pain [16]. Similar results were obtained in a Japanese study (30.7%) [18]. Niklasson et al. [19] found PPP at 3, 6, and 12 months in 40.27 and 22% of patients, respectively, and Borges et al. [20] in 25.5% of patients at 3 months after CS. In turn, in a Chinese study, the incidence of PPP at the same intervals at 3, 6, and 12 months after CS was 18.3%, 11.3%, and 6.8%, respectively [17]. According to Kainu et al. [21], the incidence of PPP at 1 year after CS was greater (22%) than after vaginal delivery (8%). The wide variability in the reported incidence of PPP is observed. The trajectory to baseline recovery pain has not been established and ranges from two to six months, but the overall trend in the incidence of PPP is reduced after 6 months [8,15,21]. The pooled incidence of chronic postsurgical pain, according to Wang et al. [22], was 15.2% at 3 months, 9.5% at 6 months, and 5.0% at 12 months after CS, with lower incidence in low- and middle-income countries than in high-income countries.

Pain is impacting multiple domains of quality of life in more than half of women after CS [23]. We found that PPP was usually mild in nature and had little to moderate impact on function. The onset of pain after CS adversely affected daily activities, physical activity, sexual intercourse, sleep, and childcare in 84.69%, 77.30%, 61.74%, 48.47%, and 38.26% of women in the study group. In the Polish study, problems with usual activities (60%), mobility (over 50%), and self-care (33%) were similarly frequent [24]. Most women with PPP reported mild pain (NRS 1–4), which is confirmed by Jin et al. [17]. Moderate pain (NRS 4–6) predominated in the Swedish study [19]. Borges et al. [20] reported that the most intense persistent pain was rated by 16.1%, 47.5%, and 36.4% of respondents as mild, moderate, and severe pain, respectively, while the mean pain intensity was 5.7 (2.3 SD) on an NRS scale. In the present study, mean PPP intensity was lower (3.44/1.83 SD).

When analyzing the issue of persistent pain after cesarean section, the terms wound pain and non-wound pain are used [15]. Overall estimated incidence of wound pain at 3 to less than 6 months after CS is 15.4%, and at 12 months after CS it is 11.5% [15]. In our own study, the postoperative scar was indicated as the location of pain in almost every second woman (44.13%). Similar results were obtained by Jin et al. [17]. Niklasson et al. [19] reported that 56% of all responders with pain reported it in and around the surgical site. The Pfannenstiel incision frequently used for CS is associated with a risk of neuropathic pain as a result of ilioinguinal and iliohypogastric nerve entrapment [5]. The neuropathic component was found in every fourth woman with chronic scar pain [25]. In our own study, the stabbing and burning pain was reported by 151/392 (38.52%) and 60/392 (15.31%) women with PPP.

Among non-wound pain, pelvic and back pain are the most common, sometimes referred to as lumbopelvic pain. According to Weibel et al. [15], the pooled incidence rates of chronic back pain at 3 to less than 6 months after CS was 29.8%, at least 12 months after CS was 17.5%, and chronic pelvic pain equaled 19.4% and 22.1%, respectively. Similar results were obtained by Niklasson et al. [19]. In turn, a Canadian study found its presence in 21% of women 6 months after CS [26]. The incidence of chronic back pain revealed in our own study was higher than the rate of postoperative wound or pelvic pain. It was reported by every second woman. The risk factors for persistent postpartum back pain are a history of low back pain, a pre-pregnancy body mass index > 25, pelvic girdle pain in pregnancy, depression in pregnancy, a heavy workload in pregnancy [27], and a heavier baby’s weight, but not spinal anesthesia [28] or higher NRS scores before pregnancy or at multiple pain sites [29].

In our own study, a history of pain during pregnancy, previous surgery, and severe acute postoperative pain soon after CS were significant risk factors for PPP. This relationship between poorly controlled acute pain after CS and persistent pain is well described [5,10,16,17,19,20]. It is the most commonly identified factor associated with PPP [30]. In addition to these, the age, weight of the woman, psychological factors (anxiety, depression), type of anesthesia, and factors related to surgery or tissue injury have so far been associated with the development of chronic pain after CS [9,20,30].

### Limitations

The retrospective and self-reported nature of the data introduces potential recall bias and selection bias. Respondents may not remember the intensity or duration of pain accurately. Subjective assessment of pain after months may be distorted by emotions, current health status, or the passage of time. The use of electronic patient-reported outcome (ePRO) questionnaires has methodological limitations. One of the main problems is the risk of selection bias resulting from unequal access to digital technologies among respondents, which may limit the representativeness of the sample. Respondents with chronic pain may be more likely to participate in the study, which may distort the results. The lack of direct contact with the researcher makes it difficult to clarify any doubts regarding the questions, which may lead to imprecise or ambiguous answers. The study did not take into account psychosocial determinants such as anxiety, depression, or catastrophizing.

## 5. Conclusions

Persistent postpartum pain is a significant problem affecting 32.37% of the study population. Pre-existing pain conditions, previous surgery, and severe postoperative pain are potential risk factors for PPP. Women hospitalized in a facility of a higher referral system reported significantly less severe postoperative pain, just like women who did not report pregnancy pain. Persistent postpartum pain is not limited only to the area of surgery. Persistent back pain onset after CS was reported by every second woman. There is a need to identify women at greater risk of developing PPP and implement individualized and preventive clinical management. The implementation of preventive perioperative protocols based on effective analgesia and postpartum care plans focused on long-term recovery may reduce the risk of PPP.

## Figures and Tables

**Table 1:** Population sample descriptive analysis (N = 1211).

Parameter		Total (N = 1211)
Age [years]	Mean (SD)	32.51 (4.22)
	Median (quartiles)	32 (30–35)
	Range	21–47
	N	1211
Number of CSs	1 CS	774 (63.91%)
	2 CSs	381 (31.46%)
	3 CSs	50 (4.13%)
	More than 3 CSs	6 (0.50%)
History of surgeries	No	866 (71.51%)
	Yes	345 (28.49%)
Pregnancy complications *	Diabetes	201 (16.60%)
	Cholestasis	20 (1.65%)
	Hypertension	198 (16.35%)
	Genital tract bleeding	106 (8.75%)
	Urinary tract inflammation	71 (5.86%)
	Cervical insufficiency	24 (1.98%)
Number of pregnancy complications	No complications	723 (59.70%)
	1 complication	368 (30.39%)
	2 complications	108 (8.92%)
	3 complications	12 (0.99%)
Level of referral system	Primary level	210 (17.34%)
	Secondary level	180 (14.86%)
	Tertiary level	605 (49.96%)
	Unknown	216 (17.84%)
Length of hospitalization	Standard (3 days)	711 (58.71%)
	Extended (newborn’s condition)	398 (32.87%)
	Extended (mother’s condition)	102 (8.42%)
Type of anesthesia	General anesthesia	139 (11.48%)
	Spinal anesthesia	1072 (88.52%)
Type of sutures	Nonabsorbable sutures	568 (46.90%)
	Absorbable sutures	643 (53.10%)
Wound drainage	No	1033 (85.30%)
	Yes	178 (14.70%)
Early skin-to-skin contact	Mother	258 (21.30%)
	Father	449 (37.08%)
	None	504 (41.62%)
Newborn feeding	Breastfeeding	449 (37.08%)
	Bottle feeding	122 (10.07%)
	Mixed feeding	640 (52.85%)
Postoperative period duration [months]	Mean (SD)	25.24 (25.8)
	Median (quartiles)	17 (7–36)
	Range	1–151
	N	1211
Postoperative period duration groups	Up to 12 months	493 (40.71%)
	13–24 months	279 (23.04%)
	25–36 months	182 (15.03%)
	37–48 months	103 (8.51%)
	49–60 months	61 (5.04%)
	Over 60 months	93 (7.68%)

* multiple-choice question—percents do not sum up to 100.

**Table 2:** Pregnancy pain characteristics.

Parameter		Total (N = 1211)
Pain during pregnancy	No	410 (33.86%)
	Yes	801 (66.14%)
Localization of pain—pregnancy *	Cervical spine	43 (3.55%)
	Thoracic spine	72 (5.95%)
	Lumbar spine	508 (41.95%)
	Pubic symphysis	296 (24.44%)
	Anal region	159 (13.13%)
	Groin region	5 (0.41%)
	Pelvic floor	138 (11.40%)
	Lower limbs	197 (16.27%)
	Upper limbs	27 (2.23%)
	Sacrococcygeal region	191 (15.77%)
	Ribs	3 (0.25%)
	Abdomen	16 (1.32%)
Number of pain localizations—pregnancy	Mean (SD)	1.37 (1.37)
	Median (quartiles)	1 (0–2)
	Range	0–7
	N	1211
Physiotherapy treatment during pregnancy	No	829 (68.46%)
	Yes	382 (31.54%)
Reason for physiotherapy *	Scar	16 (1.32%)
	Low back pain	169 (13.96%)
	Pelvic pain	44 (3.63%)
	Abdominal pain	26 (2.15%)
	Prevention	115 (9.50%)
	Urinary incontinence	7 (0.58%)
	DRA ^#^	16 (1.32%)
	Pubic diastasis	7 (0.58%)

* multiple-choice question—percents do not sum up to 100. ^#^ DRA—diastasis recti abdominis.

**Table 3:** Characteristics of postoperative pain (early stage of convalescence, during hospitalization).

Parameter		Total (N = 1211)
Pain day 0 NRS ^#^	Mean (SD)	5.44 (2.78)
	Median (quartiles)	6 (3–8)
	Range	0–10
	N	1211
Pain day 0 NRS—groups	0	80 (6.61%)
	1–4	334 (27.58%)
	5–7	465 (38.40%)
	8–10	332 (27.42%)
Pain discharge day NRS	Mean (SD)	3.6 (2.29)
	Median (quartiles)	3 (2–5)
	Range	0–10
	N	1211
Pain discharge day NRS—groups	0	108 (8.92%)
	1–4	691 (57.06%)
	5–7	338 (27.91%)
	8–10	74 (6.11%)
Pain-increasing activity *	Positional changes in lying position	667 (55.08%)
	Standing up	1037 (85.63%)
	Ambulation	300 (24.77%)
	Self-care	163 (13.46%)
	Breastfeeding	370 (30.55%)
	Coughing, sneezing, laughing	22 (1.82%)
Pain-decreasing activity *	Pharmacotherapy	919 (75.89%)
	Changing position	279 (23.04%)
	Ambulation	1 (0.08%)
	Skin-to-skin contact	306 (25.27%)
Pain localization—CS *	Postoperative wound	1074 (88.69%)
	Abdomen	572 (47.23%)
	Ribs	48 (3.96%)
	Head	25 (2.06%)
	Cervical spine	23 (1.90%)
	Shoulder	51 (4.21%)
	Thoracic spine	31 (2.56%)
	Lumbar spine	184 (15.19%)
	Upper limb	17 (1.40%)
	Lower limb	31 (2.56%)
Number of pain localizations—CS	Mean (SD)	1.7 (0.79)
	Median (quartiles)	2 (1–2)
	Range	0–7
	N	1211
Problems with newborn care	No	1026 (84.72%)
	Yes	185 (15.28%)
Problems with self-care	No	863 (71.26%)
	Yes	348 (28.74%)
Persistent postpartum pain	No	819 (67.63%)
	Yes	392 (32.37%)

* multiple-choice question—percents do not sum up to 100. ^#^ NRS—Numerical Rating Scale.

**Table 4:** The relationship between postoperative pain and the presence of pregnancy pain.

Parameter	Pregnancy Pain	N	Mean	SD	Median	Min	Max	Q1	Q3	*p*
Pain (day 0) NRS score	No	410	4.95	2.83	5	0	10	3	7	*p* < 0.001 *
	Yes	801	5.69	2.73	6	0	10	4	8	
Pain (discharge day) NRS score	No	410	3.34	2.45	3	0	10	1	5	*p* = 0.001 *
	Yes	801	3.73	2.19	4	0	10	2	5	
Pain decrease (discharge vs. day 0) NRS score	No	410	1.61	2.92	2	−10	10	0	3	*p* = 0.048 *
	Yes	801	1.96	2.8	2	−10	10	1	4	

*p*—Mann–Whitney U test, SD—standard deviation, Q1—lower quartile, Q3—upper quartile. * statistically significant (*p* < 0.05).

**Table 5:** Logistic regression model for the risk factors for postoperative pain (day 0).

Trait		Parameter	95%CI		*p*
Age [years]		0	−0.043	0.044	0.989
Number of CSs	1 CS	ref.			
	2 CSs	0.135	−0.253	0.523	0.495
	3 CSs	−0.099	−1.056	0.859	0.84
	More than 3 CSs	1.198	−1.265	3.661	0.34
History of surgeries	No	ref.			
	Yes	0.103	−0.287	0.494	0.604
Diabetes	No	ref.			
	Yes	0.173	−0.289	0.635	0.463
Cholestasis	No	ref.			
	Yes	0.149	−1.143	1.441	0.821
Hypertension	No	ref.			
	Yes	−0.569	−1.05	−0.088	0.02 *
Pain during pregnancy	No	ref.			
	Yes	0.668	0.294	1.042	<0.001 *
Level of referral system	Level I	ref.			
	Level II	−0.767	−1.324	−0.211	0.007 *
	Level III	−0.594	−1.037	−0.15	0.009 *
Length of hospitalization	Standard (3 days)	ref.			
	Extended (newborn’s condition)	0.208	−0.187	0.604	0.302
	Extended (mother’s condition)	0.12	−0.528	0.769	0.716
Type of anesthesia	General anesthesia	ref.			
	Spinal anesthesia	−0.372	−0.924	0.179	0.186
Type of sutures	Nonabsorbable sutures	ref.			
	Absorbable sutures	−0.005	−0.348	0.338	0.977
Wound drainage	No	ref.			
	Yes	−0.314	−0.806	0.177	0.209
Proper wound healing	No	ref.			
	Yes	−0.208	−1.099	0.684	0.648
Early skin-to-skin contact	Mother	ref.			
	Father	0.363	−0.12	0.845	0.14
	None	0.46	−0.021	0.941	0.061
Newborn feeding	Breastfeeding	ref.			
	Bottle feeding	−0.284	−0.891	0.324	0.36
	Mixed feeding	−0.001	−0.374	0.373	0.997
Lactation complications	No	ref.			
	Yes	−0.021	−0.371	0.328	0.904

*p*—multiple linear regression. * statistically significant (*p* < 0.05).

**Table 6:** Persistent postpartum pain (PPP) characteristics.

Parameter		Total (N = 392)
PPP intensity-NRS score	Mean (SD)	3.44 (1.83)
	Median (quartiles)	3 (2–4)
	Range	1–10
PPP characteristics *	Constant	10 (2.55%)
	Intermittent	189 (48.21%)
	Stabbing	151 (38.52%)
	Burning	60 (15.31%)
	Throbbing	38 (9.69%)
	Piercing	62 (15.82%)
PPP localization *	Postoperative scar	173 (44.13%)
	Abdomen	109 (27.81%)
	Thoracic spine	10 (2.55%)
	Head	49 (12.50%)
	Cervical spine	23 (5.87%)
	Lumbar spine	191 (48.72%)
	Pelvis	26 (6.63%)
	Groin	20 (5.10%)
Number of PPP localizations	Mean (SD)	1.53 (0.7)
	Median (quartiles)	1 (1–2)
	Range	1–5
PPP impact on daily activities	No impact	60 (15.31%)
	Little impact	208 (53.06%)
	Moderate impact	104 (26.53%)
	Significant impact	16 (4.08%)
	Prevents performance	4 (1.02%)
PPP impact on physical activity	No impact	89 (22.70%)
	Little impact	167 (42.60%)
	Moderate impact	89 (22.70%)
	Significant impact	38 (9.70%)
	Prevents performance	9 (2.30%)
PPP impact on sleep	No impact	202(51.53%)
	Little impact	110 (28.06%)
	Moderate impact	55 (14.03%)
	Significant impact	23 (5.87%)
	Prevents performance	2 (0.51%)
PPP impact on sexual intercourse	No impact	150 (38.26%)
	Little impact	119 (30.36%)
	Moderate impact	70 (17.86%)
	Significant impact	39 (9.95%)
	Prevents performance	14 (3.57%)
PPP impact on childcare	No impact	242 (61.74%)
	Little impact	108 (27.55%)
	Moderate impact	33 (8.42%)
	Significant impact	8 (2.04%)
	Prevents performance	1 (0.25%)
PPP-increasing activity *	Physical activity	64 (16.33%)
	Standing position	21 (5.36%)
	Lying position	28 (7.14%)
	Transfers	45 (11.48%)
	Lifting of objects	92 (23.47%)
	Leaning	33 (8.42%)
	Touch	10 (2.55%)
	Sexual intercourse	13 (3.32%)
	Menstruation	8 (2.04%)

* multiple-choice question—percents do not sum up to 100.

**Table 7:** Presence of persistent postpartum pain (PPP) in relation to postoperative period duration.

PPP	Postoperative Period Duration					
	Up to 12 Months (N = 493)	13–24 Months (N = 279)	25–36 Months (N = 182)	37–48 Months (N = 103)	49–60 Months (N = 61)	Over 60 Months (N = 93)
No	317 (64.30%)	204 (73.12%)	121 (66.48%)	70 (67.96%)	43 (70.49%)	64 (68.82%)
Yes	176 (35.70%)	75 (26.88%)	61 (33.52%)	33 (32.04%)	18 (29.51%)	29 (31.18%)

**Table 8:** Intensity of persistent postpartum pain (PPP).

PPP Intensity NRS Score	Postoperative Period Duration					
	Up to 12 Months (N = 176)	13–24 Months (N = 75)	25–36 Months (N = 61)	37–48 Months (N = 33)	49–60 Months (N = 18)	Over 60 Months (N = 29)
Mild (1–4)	145 (82.39%)	56 (74.67%)	45 (73.77%)	19 (57.58%)	10 (55.55%)	21 (72.42%)
Moderate (5–7)	28 (15.91%)	16 (21.33%)	15 (24.59%)	12 (36.36%)	7 (38.89%)	5 (17.24%)
Severe (8–10)	3 (1.70%)	3 (4.00%)	1 (1.64%)	2 (6.06%)	1 (5.56%)	3 (10.34%)

**Table 9:** Persistent postpartum pain (PPP) characteristics—localization and postoperative period duration relationship.

PPP Localization	Postoperative Period Duration					
	Up to 12 Months (N = 176)	13–24 Months (N = 75)	25–36 Months (N = 61)	37–48 Months (N = 33)	49–60 Months (N = 18)	Over 60 Months (N = 29)
Postoperative scar	92 (52.27%)	38 (50.67%)	20 (32.79%)	11 (33.33%)	3 (16.67%)	9 (31.03%)
Abdomen	45 (25.57%)	21 (28.00%)	22 (36.07%)	8 (24.24%)	6 (33.33%)	7 (24.14%)
Thoracic spine	4 (2.27%)	3 (4.00%)	1 (1.64%)	0 (0.00%)	2 (11.11%)	0 (0.00%)
Head	16 (9.09%)	4 (5.33%)	10 (16.39%)	7 (21.21%)	6 (33.33%)	6 (20.69%)
Cervical spine	8 (4.55%)	3 (4.00%)	3 (4.92%)	2 (6.06%)	2 (11.11%)	5 (17.24%)
Lumbar spine	75 (42.61%)	35 (46.67%)	31 (50.82%)	23 (69.70%)	12 (66.67%)	15 (51.72%)
Pelvis	10 (5.68%)	6 (8.00%)	3 (4.92%)	4 (12.12%)	1 (5.56%)	2 (6.90%)
Groin	15 (8.52%)	1 (1.33%)	1 (1.64%)	2 (6.06%)	1 (5.56%)	0 (0.00%)

**Table 10:** Self-assessment of the current health status.

Parameter		PPP		*p*
		No (N = 819)	Yes (N = 392)	
Current health status	Mean (SD)	80.43 (16.74)	69.29 (20.08)	*p* < 0.001 *
	Median (quartiles)	80 (75–90)	75 (60–80)	
	Range	4–100	2–100	
	n	819	392	
Current health status—groups	0–25	16 (1.95%)	20 (5.10%)	*p* < 0.001 *
	26–50	48 (5.86%)	55 (14.03%)	
	51–75	162 (19.78%)	136 (34.69%)	
	76–100	593 (72.41%)	181 (46.17%)	

*p*—qualitative variables: chi-squared or Fisher’s exact test. Quantitative variables: Mann–Whitney test. * statistically significant (*p* < 0.05).

**Table 11:** Multiple logistic regression model for the risk factors for PPP after CS.

Trait		N	n	OR	95%CI		*p*
Age [years]		-	-	0.982	0.952	1.012	0.238
Number of CSs	1 CS	774	239	1	ref.		
	2 CSs	381	134	1.296	0.984	1.707	0.065
	3 CSs	50	18	1.2	0.639	2.252	0.571
	More than 3 CSs	6	1	0.279	0.03	2.614	0.264
History of surgeries	No	866	258	1	ref.		
	Yes	345	134	1.487	1.133	1.951	0.004 *
Pain during pregnancy	No	410	105	1	ref.		
	Yes	801	287	1.481	1.126	1.948	0.005 *
Pain (day 0)		-	-	1.166	1.096	1.239	<0.001 *
Pain decrease (discharge vs. day 0)		-	-	0.879	0.829	0.933	<0.001 *
Length of hospitalization	Standard (3 days)	711	213	1	ref.		
	Extended (newborn’s condition)	398	143	1.521	1.156	2.001	0.003 *
	Extended (mother’s condition)	102	36	1.284	0.816	2.019	0.28
Type of anesthesia	General anesthesia	139	47	1	ref.		
	Spinal anesthesia	1072	345	0.953	0.645	1.41	0.81
Type of sutures	Nonabsorbable sutures	568	189	1	ref.		
	Absorbable sutures	643	203	0.968	0.756	1.241	0.8
Drainage	No	1033	325	1	ref.		
	Yes	178	67	1.327	0.939	1.874	0.109
Proper wound healing	No	46	16	1	ref.		
	Yes	1165	376	1.081	0.567	2.059	0.813

*p*—multiple logistic regression. N—group size, n—cases of chronic pain. * statistically significant (*p* < 0.05).

## Data Availability

The raw data supporting the conclusions of this article will be made available by the authors on request.
